# Increased Ammonium Enhances Suboptimal-Temperature Tolerance in Cucumber Seedlings

**DOI:** 10.3390/plants12122243

**Published:** 2023-06-07

**Authors:** Chao Ma, Tiantian Ban, Hongjun Yu, Qiang Li, Xiaohui Li, Weijie Jiang, Jianming Xie

**Affiliations:** 1 College of Horticulture, Gansu Agricultural University, Lanzhou 730070, China; 2 Institute of Horticulture, Guizhou Academy of Agricultural Science, Guiyang 550006, China; 3 Institute of Vegetables and Flowers, Chinese Academy of Agricultural Sciences, Beijing 100081, China

**Keywords:** cucumber seedlings, nitrogen forms, nitrogen uptake-transport, suboptimal-temperature tolerance

## Abstract

Nitrate nitrogen (NO_3_^−^-N) is widely used in the cultivation of the cucumber (*Cucumis sativus* L.). In fact, in mixed nitrogen forms, partially substituting NO_3_^−^-N with NH_4_^+^-N can promote the absorption and utilization of nitrogen. However, is this still the case when the cucumber seedling is vulnerable to the suboptimal-temperature stress? It remains unclear as to how the uptake and metabolism of ammonium affect the suboptimal-temperature tolerance in cucumber seedlings. In this study, cucumber seedlings were grown under suboptimal temperatures at five ammonium ratios (0NH_4_^+^, 25%NH_4_^+^, 50%NH_4_^+^, 75%NH_4_^+^, 100%NH_4_^+^) for 14 days. Firstly, increasing ammonium to 50% promoted the growth and root activity and increased protein and proline contents but decreased MDA content in cucumber seedlings. This indicated that increasing ammonium to 50% enhanced the suboptimal-temperature tolerance of cucumber seedlings. Furthermore, increasing ammonium to 50% up-regulated the expression of the nitrogen uptake-transport genes *CsNRT1.3*, *CsNRT1.5* and *CsAMT1.1*, which promoted the uptake and transport of nitrogen, as well as the up-regulation of the expression of the glutamate cycle genes *CsGOGAT-1-2*, *CsGOGAT-2-1*, *CsGOGAT-2-2*, *CsGS-2* and *CsGS-3*, which promoted the metabolism of nitrogen. Meanwhile, increased ammonium up-regulated the expression of the PM H^+^-ATP genes *CSHA2* and *CSHA3* in roots, which maintained nitrogen transport and membranes at a suboptimal temperature. In addition, 13 of 16 genes detected in the study were preferentially expressed in the roots in the increasing ammonium treatments under suboptimal temperatures, which, thus, promoted nitrogen assimilation in roots to the enhance the suboptimal-temperature tolerance of cucumber seedlings.

## 1. Introduction

The growth of cucumber needs a large amount of nitrogen (N) fertilizer. Nitrate nitrogen (NO_3_^−^-N) can promote the vegetative growth of the cucumber grown under suitable temperatures [1]. Cucumbers cultivated in the spring have a good market price, but the seedling stage is vulnerable to the low temperature of the spring. Low temperature stress can be divided into chilling stress and suboptimal-temperature stress, especially as suboptimal temperatures occur more frequently for cucumber planting in south China, where low temperatures can affect the plants’ N utilization efficiency and lead to reduced yield and quality [2,3]. Increasing the ratio of ammonium nitrogen (NH_4_^+^-N) can stimulate N uptake in the crop and is an important way to increase N utilization efficiency [4,5]. Wang et al. [6] found that 25% NH_4_^+^-N could significantly increase morphological parameters, including the weight of root dry matter and root surface area in lettuce, while 50% NH_4_^+^-N did not significantly inhibit root growth in lettuce. Temperature is the main factor that affects the selective uptake of NO_3_^−^-N and NH_4_^+^-N in plants [7]. Zhang et al. (2017) [8] found that low temperatures were able to inhibit NO_3_^−^-N uptake, while a mixture of NO_3_^−^-N and NH_4_^+^-N could significantly promote N uptake under low-temperature conditions in cotton seedlings.

NH_4_^+^-N and NO_3_^−^-N uptake are mediated, respectively, via membrane-localized ammonium transporters (AMT) and nitrate transporters (NRT) in plant roots, and the energy needed for this process is provided through ATP. Narumol et al. [9] showed that compared to NH_4_^+^-N uptake and utilization, the process of NO_3_^−^-N uptake and reduction required more energy. Under low-temperature conditions, the energy supply in plants becomes insufficient and NO_3_^−^-N uptake and reduction may be inhibited, thereby causing N deficiency that further affects plant growth. NH_4_^+^ is absorbed by the roots and then transported to other parts of the plant via AMT on the cell membrane [10]. In tomato, the *AMT1.1* gene is first expressed in root hairs [11], also confirming that this gene plays a role in NH_4_^+^ uptake. NO_3_^−^ is absorbed through two NO_3_^−^-N transport systems, NRT1 and NRT2 [12]. The rate of NO_3_^−^ uptake in plants correlates well with the expression level of *NRT* [13]. Roots are important organs for nutrient uptake [14]. Roots can also alter their structures to adapt to the changes in the growth environment [15]. Previous studies on increasing NH_4_^+^ nutrition showed that within a suitable range, a higher NH_4_^+^ concentration could promote root growth and development [16,17] (Song et al., 2005; Wang et al., 2006). However, there are few reports on the effect of N on the nutrient uptake of roots under suboptimal-temperature stress.

After N is absorbed by the plant, it is used to synthesize amino acids, which need the regulation of a series of N metabolism genes. By searching and analysing genes encoding key enzymes in nitrate reduction and glutamate/glutamine cycle, Ma et al. [18] identified 18 genes that were involved in NO_3_^−^-N and NH_4_^+^-N assimilation. Their findings showed that the *CsNR-2* gene, *CsNR-3* gene, *CsNiR* gene, *CsGOGAT-1-1* gene, *CsGS-1* gene and *CsGDH-2* gene played important roles in N metabolism in cucumbers. Moreover, some substances are related to stress metabolism, including proline, proteins and sugars, and metabolites are related to osmoregulation in adverse situations. They are able to stabilize the structures of organelles and protect tissues from the damage caused by insufficient temperatures [19].

In summary, the uptake and utilization of nitrogen are closely related to temperature and that increasing the ratio of NH_4_^+^ to N could affect nitrogen uptake and utilization at optimal temperatures. However, whether increasing ammonium is beneficial for N uptake and utilization at suboptimal temperatures in cucumber seedlings was not reported. Moreover, the mechanism of how the uptake and metabolism of ammonium affects suboptimal-temperature tolerance in cucumber seedlings remains unclear. Thus, we carried out an experiment increasing NH_4_^+^ in cucumbers under suboptimal-temperature conditions by partially substituting NO_3_^−^-N with NH_4_^+^-N, and the levels of morphology, physiology, biochemistry and gene expression were analysed. Our goal was to provide a theoretical reference for appropriate N utilization in cucumbers under suboptimal-temperature conditions during the winter–spring seasons.

## 2. Results

### 2.1. The Effect of Increasing Ammonium on the Growth of Cucumber Plants

#### 2.1.1. The Growth of Cucumber Plants under OptimalTemperature Conditions

**Figure 1:**
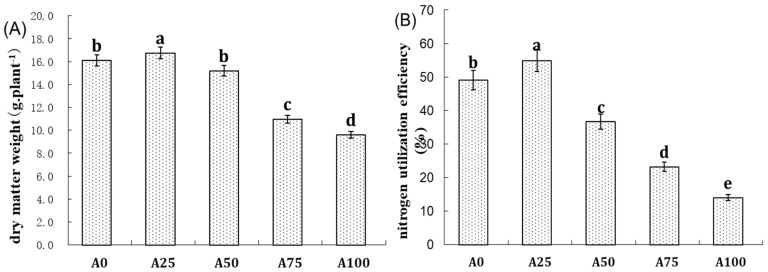
Effect of ammonium concentration on the dry matter weight and N utilization efficiency of cucumbers under optimal temperatures. (**A**) Dry matter weight; (**B**) N utilization efficiency. Bars represent the standard error, and different letters above the bars indicate significant differences at *p* < 0.05, according to Tukey’s multiple comparison test.

We studied the effect of five different compositions of NH_4_^+^-N and NO_3_^−^-N on cucumber dry weight and N utilization efficiency when grown under optimal temperatures (25 °C day/18 °C night). The A25 treatment resulted in the highest cucumber biomass (dry weight) and N utilization efficiency among five ammonium treatments, followed by A0, under optimal temperatures. A100 resulted in the lowest biomass and N utilization efficiency (Figure 1). Under optimal temperatures, cucumber biomass and N utilization efficiency were the highest under the A25 treatment followed by the A0 treatment. Figure 1A,B shows that as the ratio of NH_4_^+^ to NO_3_^−^ increased, the cucumber biomass (dry weight) and N utilization efficiency exhibited similar changes. The N utilization efficiency in the A25 treatment was higher than that in the A0 treatment; as the NH_4_^+^/NO_3_^−^ ratio increased beyond that level, N utilization efficiency significantly decreased. The biomass of cucumber in 100% NO_3_^−^-N was higher than in 100% NH_4_^−^-N; when holding the total N amount constant, substituting 25% of NH_4_^+^-N for NO_3_^−^-N resulted in further growth promotion.

#### 2.1.2. The Growth of Cucumber Plants under Suboptimal-Temperature Stress

**Figure 2:**
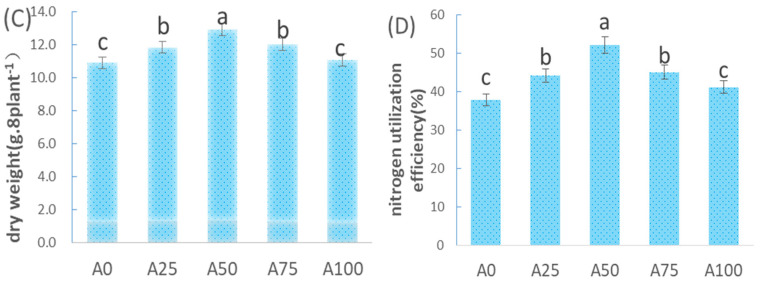
Effect of increasing ammonium on the dry matter weight and N utilization efficiency of cucumber under suboptimal-temperature stress. (**C**) Dry weight; (**D**) N utilization efficiency. Bars represent the standard error, and different letters above the bars indicate significant differences at *p* < 0.05, according to Tukey’s multiple comparison test.

While keeping the total N supply constant, using A0 (100% NO_3_^−^-N) as the control (Figure 2), NH_4_^+^ was increased to 25%, 50%, 75% or 100% under suboptimal temperatures (18 °C day/11 °C night). The dry weights and N utilization efficiency of cucumber seedlings under A50 were significantly higher than those undergoing other treatments, and those under A100 and A0 were significantly lower than those in A25, A50 and A75 (Figure 2). This showed that increasing NH_4_^+^ to 50% under suboptimal-temperature stress led to the highest biomass of cucumber plants. This result was different from the results found under optimal temperatures, where the NH_4_^+^ to N ratio of 25% promoted cucumber growth the most.

### 2.2. The Effect of Increased Ammonium on the Roots of Cucumber Plants under Suboptimal-Temperature Stress

#### 2.2.1. The Root Architecture of Cucumber Plants

**Figure 3:**
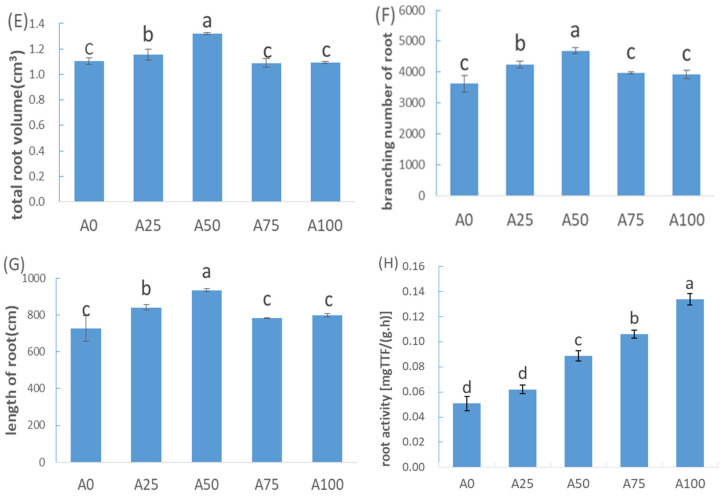
Root growth of cucumber in response to increasing ammonium under suboptimal-temperature stress. (**E**) Total root volume of each individual plant; (**F**) root forks of each individual plant; (**G**) root length of each individual plant; (**H**) root activity. Bars represent the standard error, and different letters above the bars indicate significant differences at *p* < 0.05, according to Tukey’s multiple comparison test.

**Figure 4:**
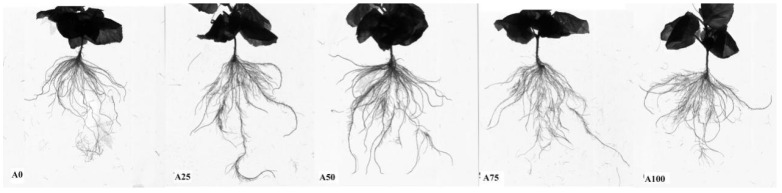
The picture of root morphology in different treatments under suboptimal-temperature stress.

The results showed that under suboptimal-temperature stress, root volume (Figure 3E), number of root branches (Figure 3F), total root length (Figure 3G) and root morphology (Figure 4) exhibited the same pattern: 0% NH_4_^+^-N < 25% NH_4_^+^-N < 50% NH_4_^+^-N. This indicated that in the 0–50% range, increasing NH_4_^+^ promoted cucumber root growth more, while in the 50–100% range, a higher ratio of NH_4_^+^ to N resulted in lower root volume, number of root branches and total root length.

Root activity measured using the TTC method was closely correlated with the number of growth points in roots and their physiological activities. Furthermore, a higher triphenyl tetrazolium formazan value of the TTC-reduced substance indicated a higher physiological activity of the root. Figure 3H showed that the cucumber root activity increased when the ratio of NH_4_^+^ was increased from 0% to 100% under suboptimal-temperature stress. Root activity was the highest in the A100 treatment, while that in A0 treatment was the lowest.

#### 2.2.2. The Root/Shoot Ratio in Cucumber Seedlings

Under suboptimal-temperature stress, the root/shoot ratio in cucumber seedlings gradually decreased when the ratio of NH_4_^+^ to N in the NO_3_^−^/NH_4_^+^ composition increased. However, increased NH_4_^+^-N did not decrease the root dry weight (Root (DW)) (Table 1).

### 2.3. The Effect of Increased Ammonium on Adversity Metabolites under Suboptimal-Temperature Stress

**Figure 5:**
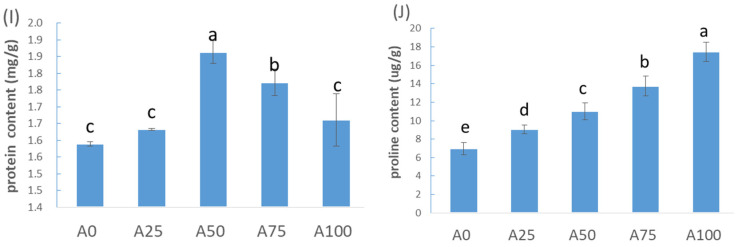

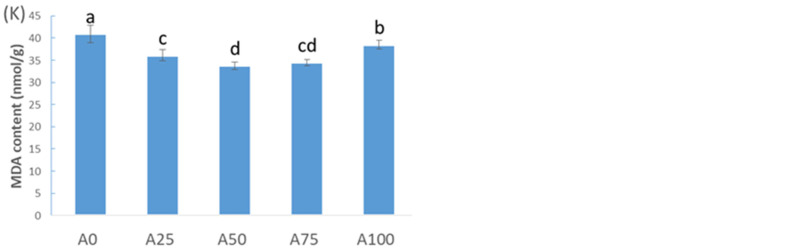
Adversity indicators of cucumber in response to increasing ammonium under suboptimal-temperature stress. (**I**) Protein content; (**J**) proline content; (**K**) MDA content. Bars represent the standard error, and different letters above the bars indicate significant differences at *p* < 0.05, according to Tukey’s multiple comparison test.

The protein content reached the highest level under A50, followed by A75, whose level was lowest under A0 (Figure 5I). The proline continuously increased when NH_4_^+^-N increased from 0% to 100% (Figure 5J). The MDA content was the lowest under A50 and the highest under A0, which was opposite to the changes in protein content (Figure 5I,K). The results show that protein content first increased, then decreased, while MDA content first decreased, then increased when the ratio of NH_4_^+^ to N increased.

### 2.4. The Effect of Increased Ammonium on Nitrogen-Related Gene Expression in Cucumber Plants under Suboptimal-Temperature Stress

#### 2.4.1. The Expression of Nitrogen Uptake-Transport Genes

**Figure 6:**
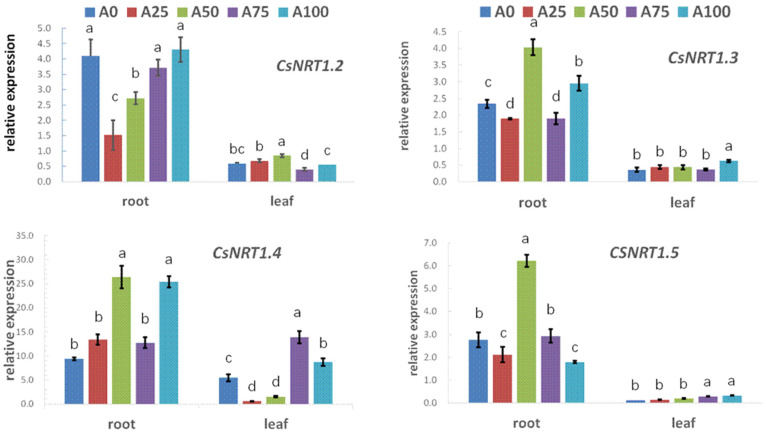

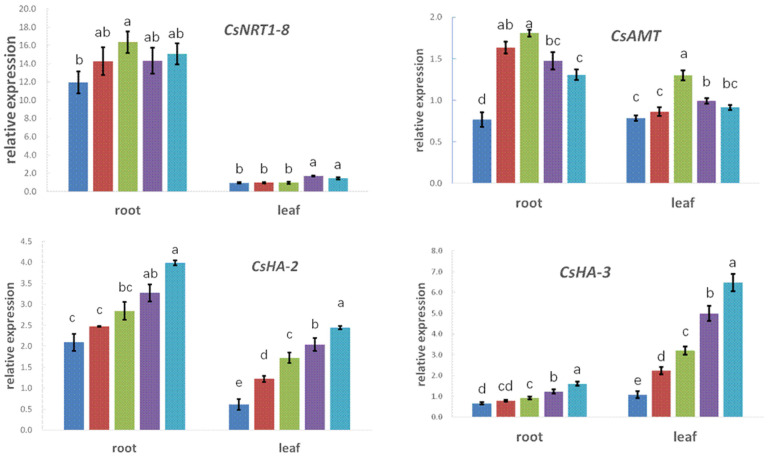
Effect of increasing ammonium on N-uptake and -transport gene expression in cucumber seedings under suboptimal-temperature stress. Bars represent the standard error, and different letters above the bars indicate significant differences at *p* < 0.05, according to Tukey’s multiple comparison test.

We used qRT-PCR to measure the relative expression levels of genes (Figure 6). The nitrate-uptake and -transport gene, *CsNRT1.2*, had its highest expression level in the leaves under A50, while *CsNRT1.3* and *CsNRT1.5* had their highest expression levels in the roots under A50. *CsNRT1.2*, *CsNRT1.3*, *CsNRT1.4*, *CsNRT1.5* and *CsNRT1.8* exhibited significantly higher expression in the roots than in the leaves. Regarding ammonium-uptake and -transport genes in roots and leaves, the expression of the NH_4_^+^-N-transport gene *CsAMT1.1* increased as the ratio of ammonium increased from 0% to 50%, while its expression decreased as the ratio of ammonium increased beyond that. Its expression level was highest under A50. Moreover, under all increased NH_4_^+^ treatments, the *CsAMT1.1* gene was expressed more highly in the roots than the leaves. Plasma membrane H^+^-ATPase genes in roots and leaves, namely the ATPase genes *CsHA2* and *CsHA3*, were expressed more highly as the ratio of NH_4_^+^ to N increased from 0% to 100%, but they displayed some differences to each other. In all treatments with increased NH_4_^+^, *CsHA2* was higher in the roots than in the leaves, while *CsHA3* was significantly lower in the roots than in the leaves.

#### 2.4.2. The Expression of Nitrogen-Metabolic Glutamine Cycle Genes

**Figure 7:**
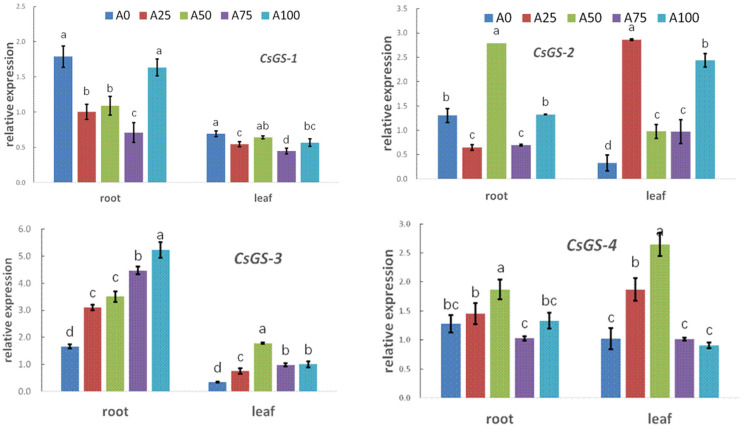

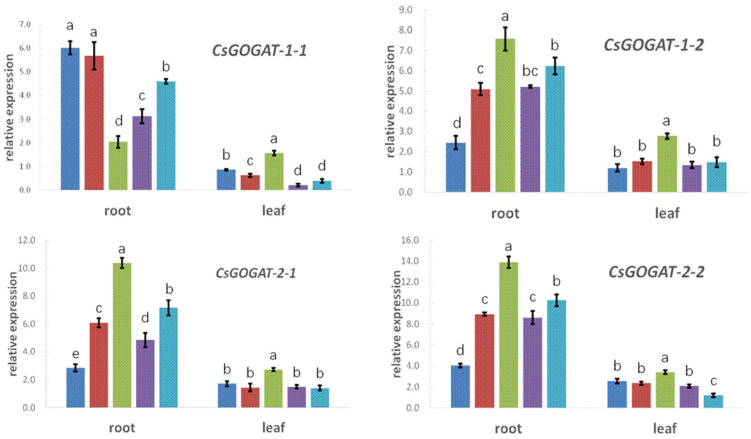
Effect of increasing ammonium on the genes of the GS/GOGAT cycle in cucumber seedings under suboptimal-temperature stress. Bars represent the standard error, and different letters above the bars indicate significant differences at *p* < 0.05, according to Tukey’s multiple comparison test.

The results showed that in the roots, *CsGS-2* had its highest expression level under A50. *CsGS-3* expression in the roots increased as the ratio of NH_4_^+^ to N increased from 0% to 100%. In the leaves, *CsGS-3* was expressed more highly, as the ratio of NH_4_^+^ to N increased from 0% to 50%, reaching its highest level in A50. Moreover, the expression level of *CsGS-3* in the roots was higher than in the leaves in all treatments with increased NH_4_^+^ (Figure 7).

The expression of *CsGOGAT-1-2*, *CsGOGAT-2-1* and *CsGOGAT-2-2* showed similar patterns in the roots of cucumber seedlings: when NH_4_^+^ increased by 0–50%, they all increased, reaching their highest levels under A50. In the leaves, *CsGOGAT-1-1*, *CsGOGAT-1-2*, *CsGOGAT-2-1* and *CsGOGAT-2-2* all exhibited their highest expression levels when the NH_4_^+^ to N ratio was 50%. However, the expression levels of these four genes in the leaves were significantly lower than those in the roots under all treatments with increased NH_4_^+^ (Figure 7).

## 3. Discussion

### 3.1. The Responses of Seedling Growth and Root Morphology to Increased Ammonium under Suboptimal-Temperature Stress

Although vegetable cultivation mainly uses NO_3_^−^-N as the N source, increasing NH_4_^+^ nutrition appropriately can not only increase vegetable yield and reduce the content of nitrate salt but also increase N utilization efficiency [20,21]. Our study found that cucumbers required a higher ratio of NH_4_^+^-N under suboptimal-temperature stress compared to optimal temperature conditions. Zhang et al. [8] also found that NO_3_^−^-N uptake in cotton seedlings was significantly inhibited by suboptimal temperatures, but this inhibitory effect was alleviated by partially substituting NO_3_^−^-N with NH_4_^+^-N. Similar results were shown in a study on N in sweet melon seedlings by Gao et al. [22]. Regarding the reason why plants require a higher ratio of NH_4_^+^ under suboptimal-temperature conditions, Di et al. [23] proposed that both NO_3_^−^ uptake-transport and NO_3_^−^ reduction require energy, a too high NO_3_^−^ to N ratio could lead to the overconsumption of energy, and plants tend to favour the uptake and assimilation of NH_4_^+^-N with less energy consumption under suboptimal-temperature stress.

This study found that under suboptimal temperatures, with the ratio of NH_4_^+^ to N increasing from 0% to 100%, cucumber root parameters first increased and then decreased, indicating that increasing NH_4_^+^ to 50% maximized root growth (Figure 3). However, the root/shoot ratio of cucumber seedlings gradually decreased (Table 1). Then, we further analysed root activity, which was increased when the ratio of NH_4_^+^ increased under suboptimal temperatures. Root activity and N uptake ability are significantly correlated [24,25]. In this study, although the treatments with increased NH_4_^+^ decreased the root/shoot ratio, the root dry weight and root volume did not significantly decrease (Table 1). Therefore, nutrient uptake via roots was strengthened and plant growth was promoted.

### 3.2. The Responses of Adversity Metabolites to Increased Ammonium under Suboptimal-Temperature Stress

The results show that the pattern of MDA content changes with the ratio of NH_4_^+^ was exactly opposite to that of protein content when the ratio of NH_4_^+^-N increased (Figure 6). These results indicated that MDA accumulation induced by suboptimal-temperature stress led to protein damage and the inhibition of the protein synthesis. MDA is the main product of membrane lipid peroxidation, and it also has inhibitory effects on protein synthesis [26]. The level of protein in plants increases under different stresses, which helps to maintain a relatively low osmotic potential in the cell [27]. Moreover, we found that the protein and proline contents in the cucumber leaves increased as NH_4_^+^ increased from 0% to 50% under suboptimal-temperature stress, which could strengthen the plant’s water retention ability and alleviate cellular damage caused by suboptimal temperatures. Thus, an increase in intracellular proline is able to maintain cell osmotic pressure and prevent excessive water loss, alleviating cell damage from low temperatures [28].

### 3.3. The Responses of Nitrogen-Related Genes to Increased Ammonium under Suboptimal-Temperature Stress

- Nitrogen uptake-transport genes

In this study, *CsNRT1.3*, *CsNRT1.5* and *CsAMT1.1* in roots had their highest expression levels in the treatment of 50% NH_4_^+^, corresponding to the leaf protein content and seedling biomass. Additionally, the expression of seven nitrogen uptake-transport genes was higher in roots than those in leaves (Figure 6). This indicated that during this stage, when NH_4_^+^ was high, the main action site of these genes was the root. Tang et al. [29] and Forde et al. [30] reported the specific gene expression patterns of NO_3_^−^-N-uptake and -transport proteins in different tissues of crops. In addition, we also found that the expression of *CsNRT1.3* and *CsNRT1.5* in the roots was higher than under other conditions when NH_4_^+^ increased to 50%, indicating that the NH_4_^+^ to N ratio of 50% promoted the transport of NO_3_^−^-N from roots to shoots. Migocka et al. [13] found that *CsNRT1.3* and *CsNRT1.5* transported NO_3_^−^-N from roots to shoots.

The *AMT* gene family contains *AMT1* and *AMT2*. The *AMT1* gene plays an important role in NH_4_^+^ uptake in the roots from the soil [31]. In *Arabidopsis*, the ammonium uptake ability can be altered by the changes in the expression of the *AtAMT1.1*, *AtAMTl.2* and *AtAMTl.3* genes [32]. In this study, we found that the NH_4_^+^-N transporter gene *CsAMT1.1* in the roots and leaves of cucumber seedlings was first upregulated and then downregulated with the increase in the NH_4_^+^ to N ratio. These results indicate that the *CsAMT1.1* gene was induced by NH_4_^+^-N, but a higher NH_4_^+^ to N ratio was not necessarily better: the 50% NH_4_^+^ to N ratio induced the upregulation of *CsAMT1.1*, thereby promoting NH_4_^+^-N uptake and transport in cucumber seedlings.

Our study found that the root PM H^+^-ATPase genes *CsHA2* and *CsHA3* were upregulated as the NH_4_^+^-N ratio increased from 0% to 100%, indicating that increasing NH_4_^+^ elevated the expression of the plasma membrane H^+^-ATP genes. Miguel et al. [33] reported that H^+^-ATP was very important in the secondary transmembrane transport of nutrients, especially plant cells involved in environmental stress. It is possible that NH_4_^+^-N induced the depolarization of the cell membrane, which then activated the plasma membrane H^+^-ATPase and induced the expression of the *CsHA2* and *CsHA3* genes [34,35].

- Nitrogen metabolism genes

The expression of *GS* and *GOGAT* genes reflect the level of N metabolism to a certain extent [36]. Our study found that in the roots, *CsGS-2* expression was at its highest under the 50% NH_4_^+^ treatment. The expression of *CsGS3* in roots was increased when the ratio of NH_4_^+^ to N increased. These data showed that the expression levels of two genes involved in the same process exhibited different response to the ratio of NH_4_^+^, but treatments increasing NH_4_^+^ to a suitable level could upregulate the expression of both genes. In addition, our study showed that upon suboptimal-temperature stress, the expression of *CsGOGAT-1-2*, *CsGOGAT-2-1* and *CsGOGAT-2-2* in the leaves and roots all reached their highest levels when the NH_4_^+^ to N ratio was 50% (Figure 7). The *CsGOGAT* gene family exhibited good consistency in response to increased NH_4_^+^, indicating that increasing NH_4_^+^ to 50% most significantly induced their expression. Summarizing the above analysis of N metabolism genes, after the exogenous N was absorbed and transported, via the glutamine–glutamate cycle regulated by the *CsGS-2*, *CsGS-3*, *CsGOGAT-1-2*, *CsGOGAT-2-1* and *CsGOGAT-2-2* genes, amino acids and other N compounds necessary for plants were synthesized.

Moreover, the expression levels of six genes in the roots were significantly higher than those in the leaves in all treatments with increased NH_4_^+^. We supposed that a large amount of NH_4_^+^ was rapidly assimilated upon entering the plant roots in order to be transported into the leaves for plant growth. As a result, they played an important role in promoting nitrogen assimilation to enhance the suboptimal-temperature tolerance of the cucumber seedlings.

**Figure 8:**
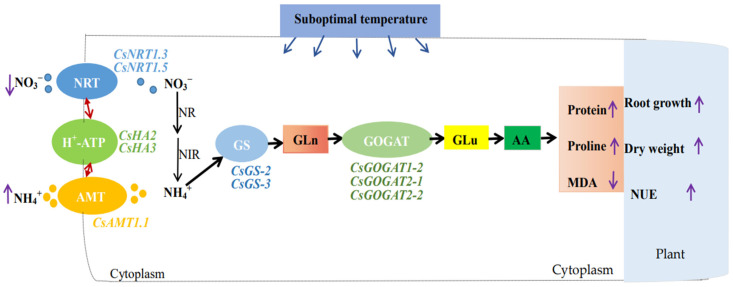
Schematic diagram of increasing ammonium to reduce the growth inhibition of cucumber seedlings under suboptimal-temperature stress. NO_3_^−^- and NH_4_^+^- uptake are mediated from the extracellular space to the cytosol via a nitrate transporter and an ammonium transporter in collaboration with H^+^-pumping ATP, respectively. After that, NO_3_^−^ is reduced to NH_4_^+^ and NH_4_^+^ undergoes the GS/GOGAT cycle to synthesize amino acids, which are then converted into other components required by plants.↑: increase;↓: diminish; ↕: interaction; NRT: nitrate transporter; AMT: ammonium transporter; H^+^-ATP: H^+^-pumping ATPase; GLn: glutamine; GLu: glutamate; AA: amino acid; MDA: malondialdehyde; NUE: nitrogen utilization efficiency.

Through the above research and discussion, we proposed schematic diagram of increasing ammonium to reduce the growth inhibition of cucumber seedlings under suboptimal-temperature stress (Figure 8).

## 4. Materials and Methods

### 4.1. Materials and Plant Growth Conditions

This experiment was carried out in the greenhouse and growth chambers at the Guizhou Academy of Agricultural Sciences, China (26°35′ N, 106°42′ E), in 2020. The experimental cucumber variety was ‘Zhongnong No. 26′, selectively bred at the Institute of Vegetables and Flowers, Chinese Academy of Agricultural Sciences. Cucumber seeds were soaked in warm water, sterilized, wrapped in cotton gauze and germinated in the dark at 28 °C for 24 h. Germinated seeds were then sown in medium dishes (54 holes) filled with vermiculite. The cucumber seedlings were transferred to a liquid medium box filled with 1/2 standard Hongland nutrients (EC = 2.4–2.6 ms/cm) when two leaves appeared. The treatments were begun at the four-leaf stage of the cucumber seedlings, which were then cultivated for 14 days.

### 4.2. Experimental Treatments

(1) Temperature treatment: Suboptimal-temperature stress for cucumber was 18 °C (day)/11 °C (night). Optimal temperature was 28 °C (day)/21 °C (night).
(2) Nitrogen source treatment: Treatment with different N sources was carried out using different liquid media for cucumber cultivation. The molar quantity of N remained the same in all liquid media (12 mmol/L), while the N forms were different. The liquid medium base was a new formulation developed based on the Hongland formulation [37] by adjusting the N forms.

Full NO_3_^−^-N (100% NO_3_^−^) was used as the control, and different ratios of NH_4_^+^-N substitution were the treatments (Table 2). In the table, A represents NH_4_^+^-N (ammonium), and the percentages (%) represent the ratios of NH_4_^+^ to N and NO_3_^−^ to N out of the total N. Five treatments with three biological replicates were applied to a total of 90 cucumber seedlings in the experiment; thus, six cucumber seedlings were collected per treatment per replicate.

### 4.3. Methods

#### 4.3.1. Measurements of Plant Biomass

Cucumber plants were harvested 14 days after the suboptimal-temperature stress. After harvest, plants were washed with distilled water and separated into two parts, namely shoots and roots. Dry weight was measured after drying the plants in the oven at 105 °C for 20 min, followed by drying at 80 °C for 48 h.

#### 4.3.2. Measurement of Root Parameters

After harvest, roots were placed in water in the organic glass dish of the root scanner. The main root and lateral roots were spread out according to the growth position of the cucumber roots; the roots were then scanned using a root scanner (Epson G101, Seiko Epson, Naganoken, Japan). The digital software Win RHIZO 2009 was used for analysis and to obtain root parameters, including root length, root surface area, root volume and number of root tips. The number of root tips referred to the total number of root tips from all levels in the root system, while the root length referred to the total length of the main root plus all root branches.

#### 4.3.3. Measurement of Root Activity

Samples for the measurement of root activity were selected from white, newly developed root tips. A total of 0.2 g of root tips was weighed, and the triphenyltetrazolium chloride (TTC) method was used [38]. Root activity measured by the TTC method was represented by dehydrogenase activity.

#### 4.3.4. Analysis of N Utilization Efficiency

Nitrogen content in plants (g·plant^−1^ DW) was calculated as (leaf dry weight × N content per unit leaf weight) + (stem dry weight × N content per unit stem weight) + (root dry weight × N content per unit root weight).

The amount of N application in plants equalled the N content added to the liquid medium.

N utilization efficiency = N content in plants/amount of N applied [5].

#### 4.3.5. Biochemical Analysis

The third leaf counted from the top at 14 days was taken from each cucumber plant, immediately frozen in liquid N and stored at −80 °C for biochemical analyses. Protein content was measured using kits using the BCA method. Proline content was measured using the acid ninhydrin colorimetric assay [39]. Malondialdehyde (MDA) content was measured using the thiobarbituric acid method [28].

### 4.4. Total RNA Isolation and cDNA Synthesis

The total RNA of the leaves from cucumber plants was extracted using a commercial RNA extraction kit (Tiangen, Beijing, China), according to the manufacturer’s instructions. cDNA was synthesized using the Prime Script RT Reagent Kit (TaKaRa, Dalian, China), according to the manufacturer’s instructions. Finally, cDNA was used for quantitative real-time PCR (qRT-PCR) analysis.

### 4.5. Gene Expression Analysis Using qRT-PCR

To detect the gene expression levels of N-related genes in cucumbers, we utilized 9 reported genes of nitrogen uptake-transport in cucumbers and referenced the gene sequences in their experiment, including nitrate-uptake and -transport genes [13], ammonium-uptake and -transport genes [40] and plasma membrane H^+^-ATPase genes [31]. Meanwhile, we identified 8 genes involved in nitrogen metabolism in the cucumber, then clarified their sequences in previous experiments, including in *CsGS* genes and *CsGOGAT* genes [18] (Table 3). The cucumber actin gene was used as an internal control to normalize the data (accession no. XM_004147305.3) [40]. Primers were designed using Primer Premier 5 software (Primer Co., Waterloo, ON, Canada), according to the acquired gene sequences. The primer sequences used for qRT-PCR are shown in Table 4.

### 4.6. Data Analysis

Tukey’s randomized block analysis of variance (ANOVA) was used for the analysis of different N forms with the significance cut-off of *p* < 0.05. Each treatment was analysed three times. Pearson’s correlation analysis was conducted using SPSS 22.0 software (version 22.0, International Business Machines Corporation, Armonk, New York, NY, USA).

## 5. Conclusions

This study conducted experiments to investigate the effect of exogenous N forms on N uptake and metabolism under suboptimal temperatures in cucumber seedlings. The results were as follows: first of all, the most suitable ratio of NH_4_^+^ to N for the growth of cucumber seedlings under suboptimal-temperature stress was 50%, higher than the ratio (25%) under optimal temperatures. Cucumber seedlings in the 50% NH_4_^+^ treatment had the highest root parameters and the corresponding biomass and protein and proline contents but the lowest MDA content. These results indicated that increasing NH_4_^+^ nutrition to 50% elevated the tolerance of cucumber seedlings to suboptimal temperatures. Furthermore, under suboptimal-temperature stress, the level of upregulated genes involved in N uptake and metabolism was the highest when the NH_4_^+^ ratio was 50%, which revealed that increased NH_4_^+^ regulated the expression of these genes, contributing to suboptimal-temperature tolerance. These genes included the nitrogen-uptake genes *CSNRT1.3*, *CSNRT1.5* and *CSAMT1.1* and glutamate cycle genes *CsGOGAT-1-2*, *CsGOGAT-2-1*, *CsGOGAT-2-2*, *CsGS-2* and *CsGS-3*. The expression of two plasma membrane H^+^-ATPase genes, *CsHA2* and *CsHA3*, increased as the NH_4_^+^ to N ratio increased from 0% to 100%. Additionally, 13 of 16 genes detected in the study had significantly higher expression levels in the roots than in the leaves. The significant response of these genes in the roots could have led to rapid N assimilation.

## Figures and Tables

**Table 1:** Effect of increasing ammonium on the root/shoot ratio of cucumber seedings under suboptimal-temperature stress.

Treatment	Root (g·plant^−1^ DW)	Shoot (g·plant^−1^ DW)	Root/Shoot Ratio
A0	26.4 ^c^	111.2 ^c^	0.2374 ^a^
A25	28.0 ^b^	122.8 ^b^	0.2280 ^b^
A50	30.4 ^a^	138.0 ^a^	0.2203 ^c^
A75	26.8 ^c^	126.6 ^b^	0.2117 ^d^
A100	23.5 ^d^	115.4 ^c^	0.2036 ^d^

Different letters after the numbers indicate significant differences at *p* < 0.05, according to Tukey’s multiple comparison test.

**Table 2:** The composition of nitrogen treatments in terms of different nitrogen forms.

Treatment Code	Composition of Nitrogen Forms
A0 (CK)	100% NO_3_^−^
A25	75% NO_3_^−^+25% NH_4_^+^
A50	50% NO_3_^−^+50% NH_4_^+^
A75	25% NO_3_^−^+75% NH_4_^+^
A100	100% NH_4_^+^

A: ammonium nitrogen; %: the percentage of NH_4_^+^-N or NO_3_^−^ to the total nitrogen.

**Table 3:** List of 17 genes related to the nitrogen metabolism of cucumber.

Gene Abbreviation	Accession No.	Function
*CsNRT1.1*	NM_001288600.1	nitrate transporter
*CsNRT1.2*	JX908737.1	nitrate transporter
*CsNRT1.3*	JX206800.1	nitrate transporter
*CsNRT1.4*	JX206801.1	nitrate transporter
*CsNRT1.5*	NM_001308933.1	nitrate transporter
*CsNRT1.8*	NM_001287472.1	nitrate transporter
*CsHA2*	NM_001305767.1	proton pump
*CsHA3*	NM_001305750.1	proton pump
*CsAMT1.1*	XM_004147130.2	ammonium transporter
*CsGS-1*	NM_001280715.1	glutamine synthesis
*CsGS-2*	XM_011661119.1	glutamine synthesis
*CsGS-3*	XM_011656924.1	glutamine synthesis
*CsGS-4*	XM_004134113.2	glutamine synthesis
*CsGOGAT-1-1*	XM_004136730.2	glutamate synthesis
*CsGOGAT-1-2*	XM_011653889.1	glutamate synthesis
*CsGOGAT-2-1*	XM_011653296.1	glutamate synthesis
*CsGOGAT-2-2*	XM_011653298.1	glutamate synthesis

**Table 4:** Primer sequences for qRT-PCRs.

Gene	Forward Primer	Reverse Primer	Length
*Actin*	5′-TCCACGAGACTACCTACAACTC-3′	5′-GCTCATACGGTCAGCGAT-3′	122 bp
*NRT1.1*	5′-TGATAGCCCTGTGCTCATTGTT-3′	5′-ACATCTCGTTCTCCCAGTTGC-3′	240 bp
*NRT1.2*	5′-TGATAGCCCTGTGCTCATTGTT-3′	5′-TGAAATCAGCCGACCCTAAA-3′	161 bp
*NRT1.3*	5′-ACTTTTCATCAGAGAAGCACCG-3′	5′-CACACAGCGAGTAGCCAATAGA-3′	168 bp
*NRT1.4*	5′-CGTTGTCACTTGGGTTCTTTG-3′	5′-GTTTGGGTTTCTGTGGCTTG-3′	240 bp
*NRT1.5*	5′-TGTTTACATTCTCAGTGTCGCAG-3′	5′-TCAGTCGCCTTTAGCATACTTTAG-3′	230 bp
*NRT1.8*	5′-GATGATGACGGAAAGGAAAGC-3′	5′-CAAAGCCAGATTGGGAGCA-3′	190 bp
*AMT1.1*	5′-GTGTCCCATTGGTTCTGGTC-3′	5′-GCCAATTCGTGGACCTTCTA-3′	168 bp
*HA2*	5′-CGAGCGTGGACTTCGATCTT-3′	5′-TGCTTTCGTCCTTGTGCTGA-3′	284 bp
*HA3*	5′-GGTTGCTACTGATGGGTGCT-3′	5′-CTTGGTCGTAAAGGCGGTCT-3′	239 bp
*GS-1*	5′-TTCTTTCTTTTGATCCAAAACCA-3′	5′-ATGTCGCCCTGTGAGACGACGCT-3′	197 bp
*GS-2*	5′-CAAGTCGGTCCTACCGTTGGTATTG-3′	5′-TCGAAGTAGACCTGTAATTGGTG-3′	188 bp
*GS-3*	5′-CTTTTGACCCCAAACCAATTCAG-3′	5′-GTGTCGACCAGTTAGACGACGCT-3′	191 bp
*GS-4*	5′-GTGCCCATCCCTACAAACAAACG-3′	5′-ACACCACAGTAATAAGGCCCCTG-3′	185 bp
*GOGAT-1-1*	5′-GAACGAGAACTTTACATTTGTAG-3′	5′-CTATATCTTCGATGATAAATAGC-3′	206 bp
*GOGAT-1-2*	5′-GAAATTGATTGAAAGAGAAGCAA-3′	5′-CTATATCTTCGATGATAAATAGC-3′	183 bp
*GOGAT-2-1*	5′-AGTTGGGATCGTGCTCAGCCT-3′	5′-CTAATTAAAAGCTCAAGAACACC-3′	216 bp
*GOGAT-2-2*	5′-ATGCGTGTTTTGGGCCACAATG-3′	5′-CTAATTAAAAGCTCAAGAACACC-3′	194 bp

## Data Availability

Data are contained within the article.
